# Sphingolipid Metabolism Remodels Immunity and Metabolic Network in the Muscle of Female Chinese Mitten Crab (*Eriocheir sinensis*)

**DOI:** 10.3390/ijms26157562

**Published:** 2025-08-05

**Authors:** Miaomiao Xue, Changyou Song, Hongxia Li, Jiyan He, Jianxiang Chen, Changxin Kong, Xiaowei Li, Hang Wang, Jie He, Pao Xu

**Affiliations:** 1 Wuxi Fisheries College, Nanjing Agricultural University, Wuxi 214081, China; 2024213003@stu.njau.edu.cn (M.X.); songchangyou@ffrc.cn (C.S.); lihx@ffrc.cn (H.L.); 2020113020@stu.njau.edu.cn (J.C.); 2023813039@stu.njau.edu.cn (C.K.); 2023113005@stu.njau.edu.cn (X.L.); 2023813063@stu.njau.edu.cn (H.W.); 2 Key Laboratory of Freshwater Fisheries and Germplasm Resources Utilization, Ministry of Agriculture and Rural Affairs, Freshwater Fisheries Research Center, Chinese Academy of Fishery Sciences, Wuxi 214081, China; 2022813053@stu.njau.edu.cn

**Keywords:** *Eriocheir sinensis*, formulated feed fattening, muscle, sphingolipid metabolism

## Abstract

Numerous studies have demonstrated the positive effects of formulated feeds on gonadal and hepatopancreatic development of *Eriocheir sinensis*. However, there are limited studies on the effects of formulated feeds on the immune homeostasis and metabolism of muscle tissue in *E. sinensis* during the fattening period. Therefore, this study used metabolomic and lipidomic to systematically analyze the effects of formulated diets on muscle metabolism in female *E. sinensis*. The results indicate that the formulated feeds improved immune performance by inhibiting inflammatory responses, apoptosis and autophagy. In addition, the feed promoted amino acid metabolism and protein synthesis while decreasing muscle fatty acid metabolism. Metabolomic analysis reveal that pyrimidine metabolism is involved in the regulation of muscle physiological health in fattening female crabs. Lipidomic analysis revealed that the formulated feeds play a role in muscle immune homeostasis, amino acid and fatty acid metabolism by regulating the level of ceramide (Cer (d18:1/22:0)) in sphingolipid metabolism. Through subnetwork analysis, the functional interactions of sphingolipid metabolism with the pathways of sphingolipid signaling, apoptosis regulation, inflammatory response and lipid dynamic homeostasis were identified, which further defined the important role of sphingolipid metabolism in the regulation of muscle physiological health and metabolic homeostasis was further identified. In summary, the formulated feeds effectively promote immune homeostasis and metabolism in the muscle of female *E. sinensis* during the fattening period. These findings provide a solid theoretical foundation for feed formulation optimization and application in fattening practices.

## 1. Introduction

*Eriocheir sinensis* (Chinese mitten crab) has become a popular delicacy in China because of its unique flavor, richness in essential fatty acids, and abundant vitamins and minerals [1,2]. According to the China Fisheries Statistics Yearbook report, the freshwater aquaculture production of *E. sinensis* reached 888,629 tons in 2023, an increase of 8.99% compared with 2022, which underscores the promising development prospects of the industry [3]. In China, *E. sinensis* usually completes pubertal molt and enters the stage of rapid growth and nutrient accumulation in the autumn of each year [4], so the use of high-quality feeds during the fattening period is crucial to enhance its growth rate and increase economic and social benefits.

In traditional aquaculture, farmers often use trash fish as fattening feed to promote the growth of *E. sinensis* for economic returns [5,6]. However, it is difficult to determine the feeding standard of trash fish, and the long-term consumption of trash fish may lead to nutritional imbalance, as well as affecting the aquaculture environment and animal health due to the perishable and pathogenic bacteria [7,8,9,10]. Therefore, many studies have been conducted to find alternative feeds that are more efficient, environmentally friendly and sustainable. Previous studies have shown that formulated diets do not negatively affect the hepatopancreas, gonads, and muscle mass of *E. sinensis* [5,11]. Instead, these diets improve growth performance and promote efficient nutrient utilization [12,13]. Similarly, our previous studies have shown that feed fattening has a positive effect on the accumulation of nutrients in the hepatopancreas and gonads of *E. sinensis* [14,15], but poor muscle fullness and prolonged market cycles of formulated feed replacement stocks still limit its widespread use [16]. Therefore, a comprehensive assessment of the impact of feed substitution on *E. sinensis* is essential to promote the sustainable development of the industry.

As consumption levels and quality of life increase, there is an increasing demand for diverse and high-quality food products. The muscles, hepatopancreas, and gonads of crabs are the primary edible parts of mature individuals. Among them, hepatopancreas and gonads have attracted much attention and become hotspots for research due to their unique taste and high nutritional value [6,17,18,19]. In contrast, relatively few studies have been conducted on muscle, despite the relatively high proportion of muscle parts in *E. sinensis*, which is certainly an area that deserves to be explored in depth. The muscles of *E. sinensis* are not only enriched with high-quality proteins, but also play an important role in the molting process. For example, muscle atrophy and recovery help protect internal organs from external environmental stresses and regulate nutrient distribution to provide the energy demands during molting [20]. Therefore, maintaining muscle health and metabolic homeostasis is essential for the optimal growth of *E. sinensis*. However, the impact of substituting trash fish with formulated feeds on muscle tissue during the fattening period in female *E. sinensis* remains unclear.

Based on the above research, this experiment aims to comprehensively evaluate the effects of formulated feeds replacement trash fish on muscle physiology, and metabolic processes during the fattening period of female *E. sinensis*. By assaying muscle enzyme activities and gene transcript levels, we investigated the role of formulated diets in regulating antioxidant capacity, immune and inflammatory responses, apoptosis and autophagy mechanisms, as well as fatty acid and amino acid metabolism. Furthermore, we explored specific changes in muscle metabolites induced by formulated feed substitution and identified key substances involved in metabolic regulation through lipidomic and metabolomic pathways. These results provide a robust theoretical foundation for the replacement of trash fish with formulated feeds in *E. sinensis* aquaculture.

## 2. Results

### 2.1. Effect of Formulated Feeds on Non-Specific Immunity

**Figure 1:**
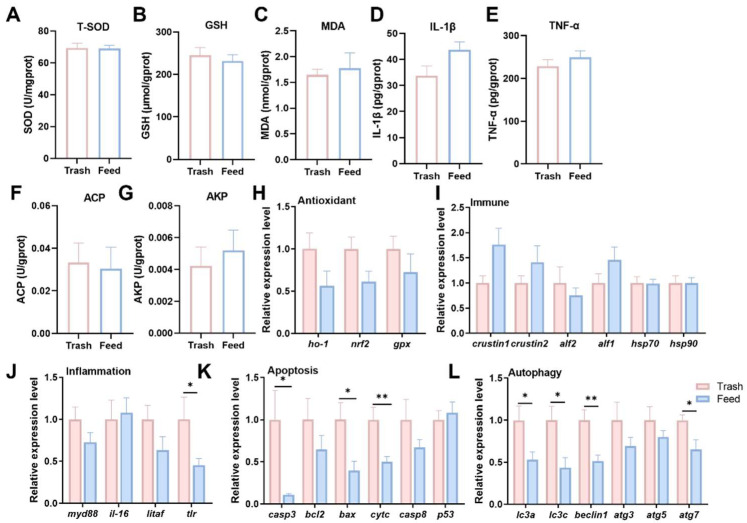
Effect of formulated feeds on non-specific immunity of fattening female *E. sinensis* (**A**), Total superoxide dismutase, T-SOD; (**B**), Glutathione, GSH; (**C**), Malondialdehyde, MDA; (**D**), Interleukin-1beta, IL-1β; (**E**), Tumor necrosis factor alpha, TNF-ɑ; (**F**), Acid phosphatase, ACP; (**G**), Alkaline phosphatase, AKP; (**H**), Expression level of antioxidant-related genes. Heme oxygenase-1, *ho-1*; nuclearerythroid-related factor 2, *nrf2*; glutathione peroxidase, *gpx*. (**I**), Expression level of immune-related genes. Anti-lipopolysaccharide 2, *alf2*; anti-lipopolysaccharide 1, *alf1*; heat shock protein 70, *hsp70*; heat shock protein 90, *hsp90*. (**J**), Expression level of inflammation-related genes. Myeloid differentiation primary response gene 88, *myd88*; interleukin 16, *il-16*; lipopolysaccharide-induced TNF-α factor, *litaf*; Toll-like receptor, *tlr.* (**K**), Expression level of apoptosis-related genes. Cysteine-aspartic acid protease 3, *casp3*; B-cell lymphoma 2, *bcl2*; Bcl-2-associated X, *bax*; cytochrome c, *cytc*; cysteine-aspartic acid protease 8, *casp8*; tumor Protein p53, *p53*. (**L**), Expression level of autophagy-related genes. Ligh Chain 3A, *lc3a*; light Chain 3C, *lc3c*; autophagy related 3, *atg3*; autophagy related 5, *atg5*; autophagy related 7, *atg7*. * *p* < 0.05 and ** *p* < 0.01. Results were indicated as mean ± SEM, *n* = 9.

Non-specific immunity is the first line of defense against exogenous stresses; therefore, studying the effects of formulated diets on the physiological health of female crab muscle is essential for assessing the feasibility of formulated diets. The results indicated that the formulated feeds did not affect the immune-related enzyme activities (Figure 1A–G). At the transcriptional level, the relative expression levels of antioxidant genes (*ho-1, nrf2, gpx*) had no statistical differences (Figure 1H), nor did the expression of immune-related genes (*crustin1*, *crustin2*, *alf1*, *alf2*, *hsp70*, *hsp90*) (Figure 1I). Inflammatory responses, cellular autophagy and apoptosis are also important adjunctive mechanisms of non-specific immunity that collectively maintain immune homeostasis. We found that the transcript levels of genes related to inflammation (*tlr*), apoptosis (*casp3*, *bax*, *cytc*), and autophagy (*lc3a*, *lc3c*, *beclin1*) were significantly decreased (*p* < 0.05, Figure 1J–L). The above findings indicate that the formulated feeds well maintain the physiological homeostasis and non-specific immunity in the muscle of female Chinese mitten crabs during the fattening period.

### 2.2. Effect of Formulated Feeds on Fatty Acid Metabolism and Protein Synthesis

**Figure 2:**
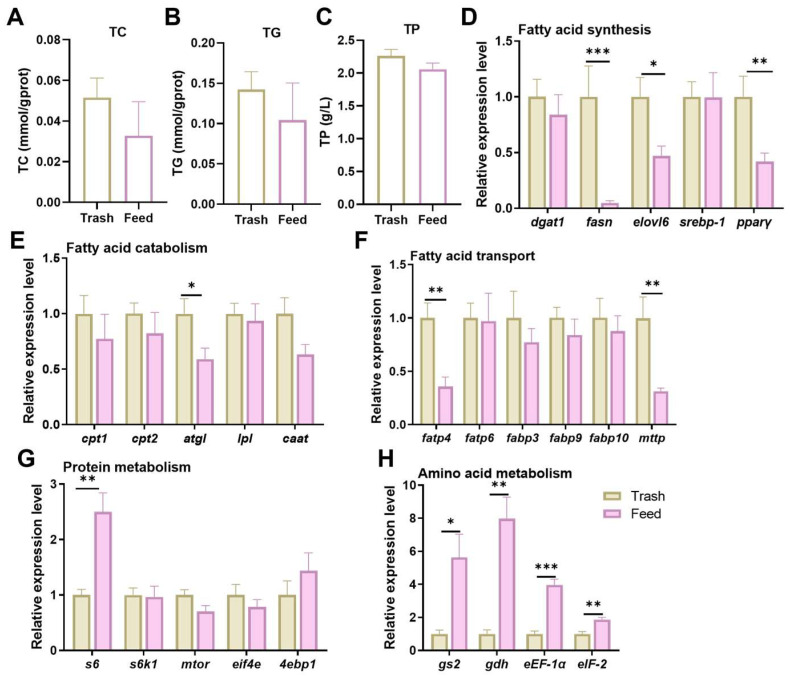
Effect of formulated feeds on fatty acid metabolism and protein synthesis of fattening female crabs. (**A**), Total cholesterol, T-CHO; (**B**), Triglycerides, TG; (**C**), Total Protein, TP; (**D**), Expression levels of fatty acid synthesis-related genes. Diacylglycerol acyltransferase 1, *dgat1*; fatty acid synthase, *fasn*; elongase of very long chain fatty acids 6, *elovl6*; sterol regulatory element-binding protein 1, *srebp-1*; peroxisome proliferators-activated receptors γ, *pparγ.* (**E**), Expression levels of fatty acid catabolism-related genes. Carnitine palmitoyltransferase1, *cpt1*; carnitine palmitoyl transferase 2, *cpt2*; adipose triglyceride lipase, *atgl*; lipoprteinlipase, *lpl*; carnitine acetyltransferase, *caat*. (**F**), Expression levels of fatty acid transport-related genes. Fatty acid transport protein 4, *fatp4*; fatty acid transport protein 6, *fatp6*; fatty acid binding protein 3, *fabp3*; fatty acid binding protein 9, *fabp9*; fatty acid binding protein 10, *fabp10*; microsomal triglyceride transfer protein, *mttp*. (**G**), Expression level of protein metabolism-related genes. Ribosomal protein S6, *s6*; ribosomal S6 protein kinase, *s6k1*; mammalian target of rapamycin, *mtor*; eukaryotic translation initiation factor 4E, *eif4e*; eukaryotic translation initiation factor 4E-binding protein 1, *4ebp1*. (**H**), Expression levels of amino acid metabolism-related genes. Glutamine Synthetase 2, *gs2*; glutamate dehydrogenase, *gdh*; eukaryotic translation elongation factor 1 α, *eEF-1α*; Eukaryotic translation initiation factor 2, *eIF-2*. * *p* < 0.05, ** *p* < 0.01 and *** *p* < 0.001. Results were indicated as mean ± SEM, *n* = 9.

To investigate the effects of formulated feed fattening on muscle metabolism in female crabs, we examined changes in muscle lipid and amino acid metabolism. For lipid metabolism, there were no remarkable changes in TC and TG content (Figure 2A,B), but feed fattening significantly inhibited the expression levels of genes related to fatty acid synthesis (*fasn*, *elovl6*, *pparγ*, *p* < 0.05, Figure 2D), catabolism (*atgl*, *p* < 0.05, Figure 2E), and transport (*fatp4*, *mttp*, *p* < 0.05, Figure 2F), indicating that the overall lipid metabolism rate was reduced. In addition, although there was no significant difference in TP content (Figure 2C), transcript levels of key genes in protein metabolism (*s6*, *p* < 0.05, Figure 2G) and amino acid metabolism (*gs2*, *gdh*, *eEF-1α*, *eIF-2*, *p* < 0.05, Figure 2H) were notably increased. These results suggest that feed fattening inhibits fatty acid metabolism while promoting amino acid metabolism in muscle.

### 2.3. Changes in Muscle Metabolites of Female E. sinensis Fatten with Formulated Feeds

**Figure 3:**
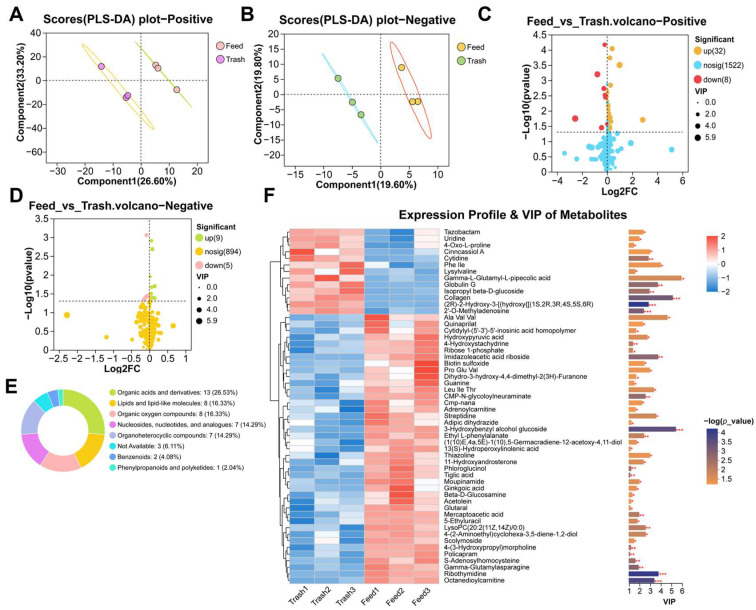
Changes in muscle metabolites of female crabs fattened on formulated feeds. (**A**,**B**), PLS-DA analysis of positive and negative metabolites; (**C**,**D**), volcano plot analysis of positive and negative metabolites; (**E**), HMDB compound classification statistics; (**F**), VIP value analysis of DEMs, the left is the metabolite clustering dendrogram and the right is the metabolite VIP histogram, with defaults >1. * *p* < 0.05, ** *p* < 0.01, and *** *p* < 0.001.

**Figure 4:**
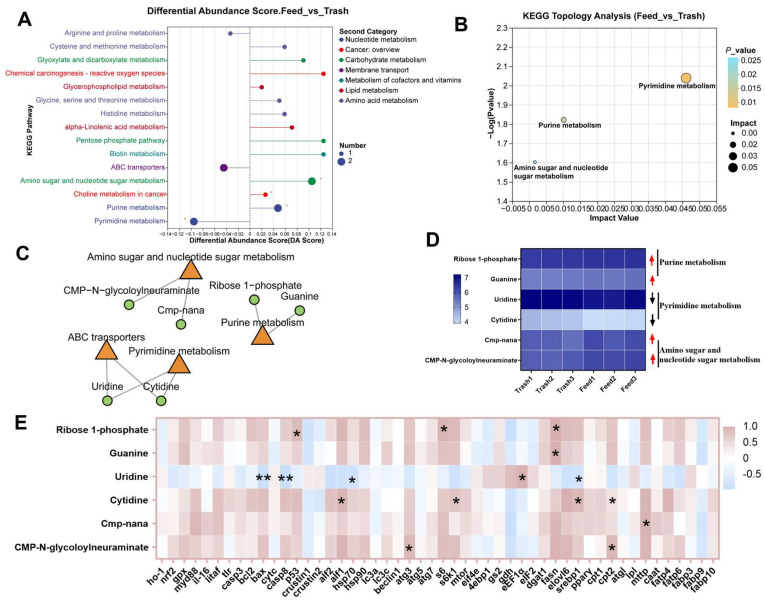
Functional enrichment and analysis of differential expressed metabolites. (**A**), KEGG pathway differential abundance score plot analysis; (**B**), KEGG topology analysis of DEMs; (**C**), KEGG enrichment analysis network diagram; (**D**), expression levels of key DEMs; (**E**), correlation analysis of key DEMs. * *p* < 0.05 and ** *p* < 0.01.

Based on the above studies, we used metabolomics to further assess the effects of feed fattening on muscle metabolism. Similarly, the results of PLS-DA showed significant differences between the two groups (Figure 3A,B), demonstrating that feed fattening affected the metabolism of female crab muscle. To explore specific changing metabolites, we performed Volcano analysis of metabolites and found that a total of 54 differential expressed metabolites (DEMs) were enriched, with 32 up-regulated and 8 down-regulated among positive metabolites, and 9 up-regulated and 5 down-regulated among negative metabolites (Figure 3C,D). The percentage of metabolites was evaluated using the HMDB and it was found that DEMs were highest in organic acids and derivatives (26.53%), followed by lipids and lipid-like molecules: 8 (16.33%) (Figure 3E). Furthermore, DEMs were divided into different subclasses (Figure 3F) and were involved in the regulation of muscle physiological health and metabolism in female crabs (Figure S1). Importantly, by KEGG differential pathway score map analysis, we found that DEMs were mainly enriched in amino acid metabolism (arginine and proline metabolism, cysteine and methionine metabolism, glycine, serine and threonine metabolism, histidine metabolism), carbohydrate metabolism (glyoxylate and dicarboxylate metabolism, pentose phosphate pathway, amino sugar and nucleotide sugar metabolism) and nucleotide metabolism (purine metabolism, pyrimidine metabolism) (Figure 4A). KEGG topology analysis showed significant enrichment of the purine metabolism, pyrimidine metabolism and amino sugar and nucleotide sugar metabolism pathways, with pyrimidine metabolism playing a more important role in the regulation of metabolism (Figure 4B). Importantly, the levels of only pyrimidine metabolism metabolites (uridine and cytidine) were significantly downregulated in these pathways (Figure 4C,D). In addition, DEMs were significantly associated with muscle immunity (*alf1*, *hsp70*), apoptosis (*bax*, *casp8*, *p53*) and autophagy (*atg3*), fatty acid metabolism (*fasn*, *srebp1*, *cpt2*, *mttp*), and protein metabolism (*s6*, *s6k1*, *eIF1α*) (*p* < 0.05, Figure 4E). The above results suggest that feed fattening regulates immune homeostasis and metabolic processes in muscle by altering the accumulation of metabolites.

### 2.4. Changes in Muscle Lipid Metabolites of Female E. sinensis Fatten with Formulated Diets

**Figure 5:**
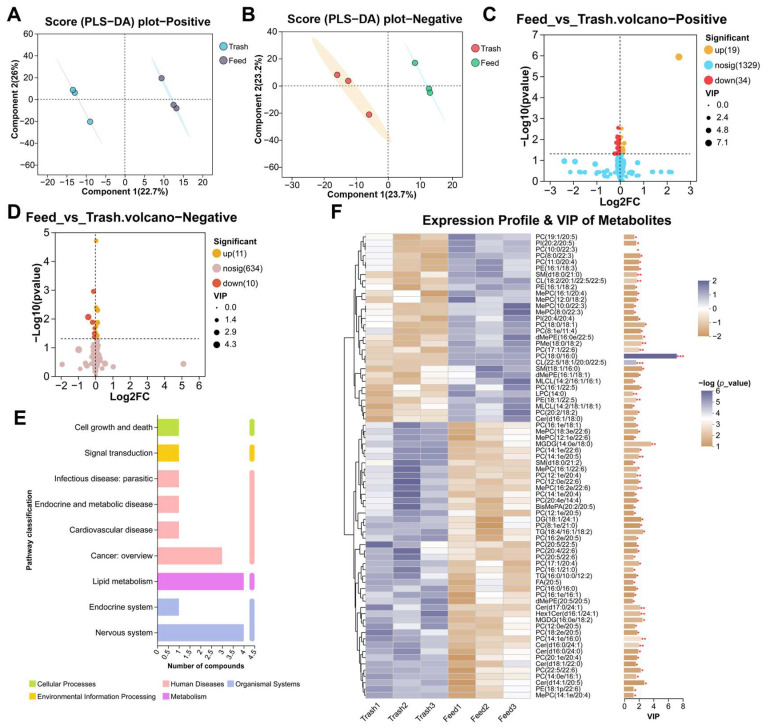
Changes in muscle lipid metabolites of female crabs fattened on formulated diets. (**A**,**B**), PLS-DA analysis of positive and negative metabolites; (**C**,**D**), volcano plot analysis of positive and negative metabolites; (**E**), KEGG metabolic pathway classification; (**F**), VIP value analysis of DLMs, the left is the metabolite clustering dendrogram and the right is the metabolite VIP histogram, with defaults >1. * *p* < 0.05, ** *p* < 0.01, and *** *p* < 0.001.

**Figure 6:**
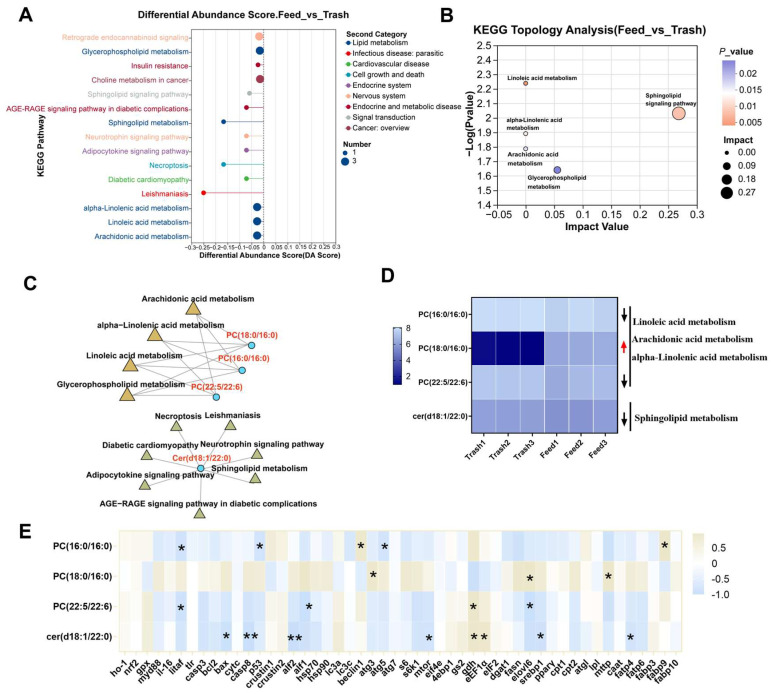
Functional enrichment and analysis of differential lipid metabolites. (**A**), KEGG pathway differential abundance score plot analysis; (**B**), KEGG topology analysis of DLMs; (**C**), KEGG enrichment analysis network diagram; (**D**), expression levels of key DLMs; (**E**), correlation analysis of key DLMs. * *p* < 0.05 and ** *p* < 0.01.

Based on the above studies, we further utilized lipidomic to explore the key lipid metabolites responsible for reduced muscle fatty acid metabolism due to formulated feed fattening. Partial least squares discriminant analysis (PLS-DA) revealed significant differences between muscle samples from different fattening modes (Figure 5A,B). In addition, a total of 74 DLMs were identified, of which 19 were up-regulated and 34 were down-regulated in positive metabolites, and 11 were up-regulated and 10 were down-regulated in negative metabolites (Figure 5C,D). KEGG metabolic pathway analysis classified these DLMs into five categories: cellular processes, environmental information processing, human diseases, organismal systems, metabolism (Figure 5E). Significant metabolites were identified by VIP analysis, which showed that DLMs were clustered into two distinct subclasses, with each metabolite contributing more than 1 to the sample variance (Figure 5F). Correlation analysis of DLMs with physiological health factors and metabolic genes revealed that most lipid metabolites are involved in physiological health and metabolic regulation (Figure S2). In addition, analysis of KEGG differential pathway score maps annotated to DLMs revealed that lipid metabolism-related pathways were mainly enriched in glycerophospholipid metabolism, sphingolipid metabolism, alpha-linolenic acid metabolism, linoleic acid metabolism, and arachidonic acid metabolism, and the expression trends of all annotated DLMs were downregulated (Figure 6A). Further analysis by KEGG topology revealed that Sphingolipid metabolism plays a more important function (Figure 6B). Interestingly, the KEGG enrichment analysis network map revealed that only four DLMs (PC (18:0/16:0), PC (16:0/16:0), PC (22:5/22:6), Cer (d18:1/22:0)) were enriched in these five pathways (Figure 6C). Importantly, only Cer (d18:1/22:0) belonged to sphingolipid metabolism (Figure 6D), and Cer (d18:1/22:0) was significantly associated with *bax*, *casp8*, *alf2*, *mtor*, *gdh*, *eEF1α*, *srebp1*, and *fatp4* genes (*p* < 0.05, Figure 6E). Based on the above findings, we hypothesized that feed fattening regulates muscle cell non-specific immunity, amino acid and fatty acid metabolism processes through the inhibition of sphingolipid metabolism (specifically Cer (d18:1/22:0)).

### 2.5. Feed Replacement of Trash Fish Inhibits Sphingolipid Metabolism to Reconfigure Muscle Tissue Metabolism and Maintain Physiological Homeostasis

**Figure 7:**
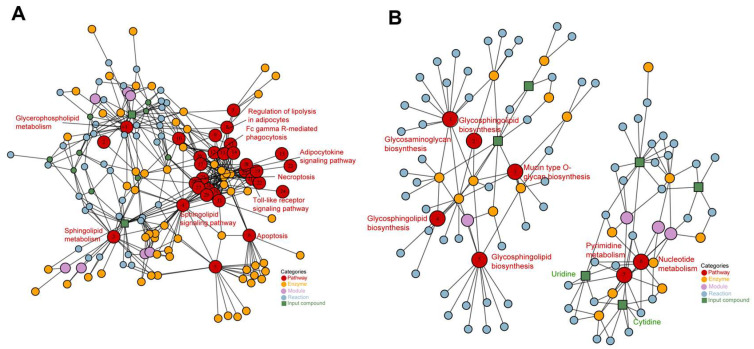
Subnetwork analysis of significantly disordered metabolic pathways. (**A**) Interactions between sphingolipid metabolic pathways and metabolites in lipidomic; (**B**) interactions between purine metabolic pathways and substances in metabolomic. Subnetwork analysis was performed using the R-based network propagation algorithm, FELLA. Nodes represent compounds, enzymes, or reactions, while edges indicate known biological interactions in KEGG.

Based on the above results, we performed a correlation analysis of the differential expressed metabolites in the lipid and metabolism groups. The results showed a highly significant correlation between sphingolipid metabolism and key metabolites in pyrimidine metabolism (*p* < 0.01, Figure S3). To further validate the relationship between differential pathways and other functional pathways identified in metabolomics and lipidomic, we applied the FELLA algorithm. In lipidomics, the analysis showed significant interactions between sphingolipid metabolism and the Glycerophospholipid metabolism, sphingolipid signaling pathway, apoptosis, Fc gamma R-mediated phagocytosis, Toll-like receptor signaling pathway, necroptosis, adipocytokine signaling pathway and regulation of lipolysis in adipocytes (Figure 7A, Table S3). This finding further supports the important role that sphingolipid metabolism may play in regulating physiological homeostasis and metabolic processes in crab muscle and is consistent with our findings described above. However, although metabolomics revealed a clear interaction between pyrimidine metabolism and nucleotide metabolism (Figure 7B), pyrimidine metabolism was not found to be directly involved in the specific metabolic pathways that regulate the physiological health of crab muscle. Unexpectedly, a significant enrichment of Glycosphingolipid biosynthesis was found in metabolomics, which is consistent with the results of the sphingolipid metabolism in the lipidomic. Taken together, this evidence suggests that the use of feed replacement of trash fish reconfigures metabolic networks in muscle tissue and maintains physiological homeostasis through targeted inhibition of sphingolipid metabolism.

### 2.6. Hypothesized Regulatory Mechanisms of Feed Replacement of Trash Fish on the Muscle of the E. sinensis

**Figure 8:**
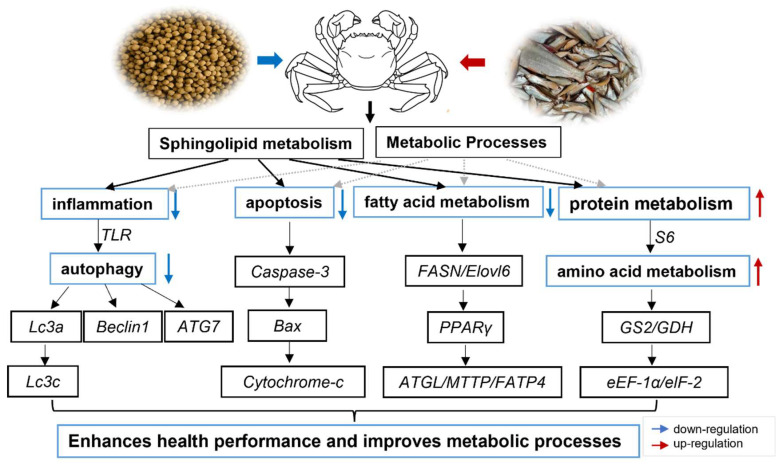
Hypothesized regulatory mechanisms of feed replacement of trash fish on the muscle of the *E. sinensis*. Red arrows indicate upregulation of gene expression levels and blue arrows indicate downregulation of gene expression levels.

Based on the above study, we revealed the potential regulatory mechanisms of formulated feed replacement of trash fish on muscle health and metabolic processes in fattening female crabs (Figure 8). Specifically, sphingolipid metabolism effectively attenuated the inflammatory response by inhibiting the TLR receptor, which in turn down-regulated the expression of key autophagy factors (e.g., LC3 family, Beclin1, ATG7). This mechanism may involve the modulation of the clearance capacity of damaged intracellular components, thus contributing to the maintenance of metabolic homeostasis. In addition, sphingolipid metabolism significantly reduced apoptosis by inhibiting the Caspase3-Bax-Cytc axis, suggesting that the feed fattening strategy was effective in reducing the occurrence of programmed cell death. Meanwhile, sphingolipid metabolism further coordinated the expression of fat metabolism (FASN/ELOVL6), lipolytic enzymes (ATGL), and lipid transporter proteins (MTTP/FATP4) through the regulation of the core transcription factor PPARγ to optimize lipid metabolic processes. Notably, sphingolipid metabolism also regulates the activity of translation initiation factors (eEF-1α/eIF-2) by affecting ribosomal protein S6 as well as amino acid metabolizing enzymes (GS2/GDH), which may have an important impact on the efficiency of protein synthesis. In conclusion, this study provides new targets for metabolic interventions in the mechanism of nutritional regulation in crustaceans and deepens our understanding of the mechanism of action of formulated dietary replacements for trash fish in the regulation of muscle health and metabolism in fattening female crabs.

## 3. Discussion

Changes in diet can lead to nutrient deficiencies or imbalances, which in turn can cause oxidative stress in aquatic animals, weaken their immune function, and even alter their cell fate, posing a serious threat to aquatic animal growth, health, and production performance [21]. Pathogen infection or inflammation can induce oxidative damage in the host, and antioxidant response can regulate immune defense and protect immune cell function [22]. As a key antioxidant enzyme, T-SOD effectively scavenges free radicals and maintains the redox balance of cells, preventing oxidative damage to muscle [23]. As an active peptide, GSH also plays a critical role in scavenging free radicals and maintaining cellular homeostasis [24]. MDA is commonly used as a marker to assess the extent of oxidative damage in living organisms [25]. *Ho-1* is a stress-responsive enzyme that protects cells from oxidative damage [26]. In addition, Gpx is involved in the maintenance of redox balance by catalyzing the reduction in hydrogen peroxide in conjunction with GSH [27]. *Nrf2* is a nuclear transcription factor that binds to antioxidant components and regulates the expression of the above-mentioned antioxidant genes [28]. The results of the present study indicated that there were no statistically significant differences in the enzyme activity and transcription levels of antioxidant factors. The probable reason for this is that the organism may have initiated adaptive adjustments to this feed change at the molecular level. When faced with exogenous or endogenous stress, the immune system triggers a series of immune responses to mitigate the harmful effects [29]. However, crustaceans lack an adaptive immune system and can only rely on innate immunity to cope with external stresses [30]. ACP and AKP play important roles in immune defense and are often used as indicators to assess changes in immune function in crustaceans [31]. In addition, antimicrobial peptides such as *alf1*, *alf2*, *crustin1*, and *crustin2* are involved in innate immune regulation and can directly eliminate or inhibit bacterial growth [32]. The heat shock proteins *hsp70* and *hsp90* are also involved in cellular antioxidant and innate immune responses [33]. This proved that formulated diet did not significantly affect the immune performance of female crab muscles. Inflammation is a typical nonspecific immune response. To further understand the potential effects of formulated feeds on muscle inflammation, we examined typical inflammatory parameters, including levels of IL-1β and TNF-α, which showed no remarkable differences in these inflammatory parameters. In addition, we examined the transcript levels of the pro-inflammatory cytokines *myd88*, *il-16*, *litaf*, and *tlr*. Notably, expression levels of *tlr* were remarkably decreased, demonstrating the potential of the formulated feed as a fattening option by suggesting that it may exert its benefits by suppressing the inflammatory response. Apoptosis is an autonomous defense mechanism of cells that can limit the spread of pathogens. Autophagy can also degrade intracellular damaged components or pathogens through lysosomes to maintain metabolic and functional homeostasis of immune cells [34]. The present study indicated that formulated feed fattening suppressed the transcript levels of apoptosis (*casp3*, *bcl2* and *cytc*) and autophagy genes (*lc3a*, *lc3c* and *beclin1*). The possible reason for this is the stable quality of the formulated feed, which avoids the carriage of pathogens by trash fish and contributes to the improvement of the cellular environment.

During the fattening stage of crabs, energy accumulation mainly depends on lipids and proteins [35]. Therefore, we investigated protein and amino acid metabolism in muscle. However, it is noteworthy that protein synthesis-related genes (*s6*) showed a significant upregulation trend, although no significant difference in TP content was observed. In addition, we were concerned that the expression of genes related to amino acids were also significantly upregulated (*gs2*, *gdh*, *eEF-1α*, and *eIF-2*), suggesting that formulated feed fattening affects amino acid metabolism and promotes protein synthesis in female crabs. Similar studies have shown that formulated feed replacement of trash fish increases the transcript levels of hepatopancreas amino acid-related genes in the *E. sinensis* [36]. The conversion of dietary lipids into body fat involves the participation of several key enzymes and transcription factors [37]. Our results showed that although formulated feed fattening did not significantly affect TC and TG content in the muscle of female crabs, this type of fattening significantly reduced the expression of genes related to fatty acid synthesis (*fasn*, *elovl6*, and *pparγ*), transport (*fatp4*), and catabolism (*atgl* and *mttp*). In contrast, our previous study showed that formulated diets promote fatty acid synthesis and inhibit their catabolism in the hepatopancreas, which in turn promotes hepatopancreatic fat accumulation [14]. This demonstrated that the hepatopancreas develops rapidly and stores large amounts of energy during fattening, in contrast to the more limited energy accumulation in muscle tissue. Studies have shown that excess dietary lipids affect crustacean metabolism and increase the risk of oxidative stress [38], so this finding has important implications for optimizing formulated feeds to enhance *E. sinensis* quality.

Metabolomic findings revealed the effects of formulated feed fattening on muscle metabolic processes in female crabs. Compared with trash fish, a total of 54 differential expressed metabolites (DEMs) were identified in the muscle tissues of female crabs fattened on formulated diets, and these metabolites were mainly enriched in amino acid metabolism, nucleic acid metabolism and lipid metabolism. In particular, pyrimidine metabolism plays a more important role in all pathways. Pyrimidine metabolism has been shown to be involved in the regulation of a wide range of cellular functions affecting the synthesis of cellular DNA and RNA, proteins, lipids and carbohydrates [39]. Uridine and Cytidine are metabolites with significant differences in the pyrimidine metabolic pathway. Research has shown that before pyrimidine metabolism can begin catabolism, Cytidine must be deaminated to Uridine by cytidine deaminase before it can be further involved in metabolism [40]. Other studies have shown that there are complex regulatory mechanisms between pyrimidine metabolism and lipid metabolism, and that lipid droplet formation may be facilitated by increasing the levels of Uridine and Cytidine in cells [39]. We hypothesize that the levels of pyrimidine metabolites regulate the rate of muscle lipid metabolism. In addition, we found that this differential expressed metabolites showed significant correlations with various aspects of the functional regulation of the immune system, apoptosis and autophagy processes, fatty acid metabolism and protein metabolism. Therefore, we hypothesized that formulated feed fattening could modulate muscle lipid metabolism as well as maintain normal physiological status and function of organismal muscle cells by altering pyrimidine metabolites.

Lipidomic analysis revealed that the differential lipid metabolites (DLMs) PC (18:0/16:0), PC (16:0/16:0), PC (22:5/22:6), Cer (d18:1/22:0) were significantly enriched in glycerophospholipid metabolism, sphingolipid metabolism, alpha-linolenic acid metabolism, linoleic acid metabolism, and arachidonic acid metabolism. Of these, PC (18:0/16:0), PC (16:0/16:0), and PC (22:5/22:6) are phosphatidylcholines, and Cer (d18:1/22:0) is an important ceramide. Phosphatidylcholine is an important component of biofilms and promotes growth in crustaceans [41]. A study by Lin et al. [42] showed that dietary supplementation with phosphatidylcholine increased hepatopancreatic antioxidant capacity and reduced lipid accumulation, but still resulted in an increase in lipid deposition in the muscle of *E. sinensis*. Similar findings were reported by Xu et al. [43], in which the fat content of *E. sinensis* muscle increased with elevated levels of phosphatidylcholine in the diet. The present study found a positive correlation between phosphatidylcholine levels and the rate of lipid metabolism, suggesting that the deposition and utilization of lipids in muscle can be controlled to some extent by modulating phosphatidylcholine levels. Ceramide (Cer (d18:1/22:0)) is one of the central products of sphingolipid metabolism, which plays an important role in cell signaling, apoptosis, and inflammatory responses [44]. During apoptosis, ceramide can act as a second messenger to activate downstream transcription factors, thereby triggering apoptosis [45]. In addition, ceramides modulate the expression of inflammation-related genes and promote the release of inflammatory factors, further exacerbating the inflammatory response [46]. In the present study, we found that the reduction in ceramide levels in the muscles of female crabs coincided with the inhibition of apoptosis and inflammatory responses. These findings suggest that by regulating the synthesis or degradation of ceramide (Cer (d18:1/22:0)) in sphingolipid metabolic pathway, we can effectively intervene in cell non-specific immune regulation, thereby improving the health status and growth performance of cultured animals.

In order to systematically analyze the integration results of metabolomic and lipidomic, this study conducted correlation analysis of all differential expressed metabolites and found that key differential expressed metabolites in sphingolipid metabolism and pyrimidine metabolism showed highly significant correlations. Based on this, we used FELLA sub-network analysis to dig deeper into the functional interactions of key metabolic pathways. Notably, metabolomics data showed significant interaction effects of glycosphingolipid metabolism, a finding consistent with the central regulatory role of sphingolipid metabolism in lipidomic. Numerous studies have shown that sphingolipids are widely distributed in skeletal muscle and are directly involved in the regulation of muscle function (apoptosis, cell proliferation, differentiation, and inflammation), whereas glycosphingolipids influence muscle metabolic homeostasis by regulating insulin sensitivity [47,48]. This finding provides a new research direction for the in-depth understanding of the regulatory network of crustacean muscle metabolism, and lays a theoretical foundation for the precise optimization of aquafeed formulations.

## 4. Materials and Methods

### 4.1. Ethics Statement

This study was conducted in accordance with the guidelines of the Animal Care and Use Commit-tee of Nanjing Agricultural University, Nanjing, China. The protocol was approved by the Animal Research Committee of Nanjing Agricultural University (permit number: SYXK (XU) 2023–0108; approved on 6 January 2023).

### 4.2. Rearing Animals and Experimental Design

After the final molt, the *E. sinensis*’ intake increases, nutrients accumulate rapidly, and the meat is fuller and tastier, which marks a critical period for fattening [49]. Before commencing the experiment, the crabs were acclimated to the outdoor culture circumstances for 3–4 days, during which they were fed trash fish. In traditional farming, trash fish served as the standard diet for the fattening stage of the Chinese mitten crabs [50]. In this study, we set the trash fish fattening group as the control group (Trash group) and the formulated feed fattening as the treatment group (Feed group). The nutritional composition of the feed is provided in Table S1. One hundred and fifty female *E. sinensis* (134.94 ± 3.56 g) that had completed their pubertal molt were obtained from the Freshwater Fisheries Research Center and randomly assigned them to six outdoor nets (20 m^3^ each, with three nets per group). Water plants necessary for the growth of *E. sinensis* and escape prevention measures were placed in the nets. Following the method described by Song et al. [14], the Trash and Feed groups were fed 8 and 4% of their body weight daily at 9:00 and 17:00 during the 5-week experimental period. During the experimental, water quality was maintained with the following parameters: DO > 5 mg/L, pH 8.1–9.0, NO_2_ < 0.02 mg/L, and NH3-N < 0.2 mg/L.

### 4.3. Sample Collection

At the end of the rearing experiment, the *E. sinensis* were starved for 24 h to evacuate the intestinal contents. Three *E. sinensis* from each net (nine per group) were randomly selected, anesthetized on ice, and then muscle tissues were collected. The tissues were rapidly frozen in liquid nitrogen and maintained at −80 °C for subsequent analysis.

### 4.4. Biochemical Index Analysis

For each group, nine muscle tissues were accurately weighed and 10% tissue homogenate (weight (g): volume (mL) = 1:9) was prepared using saline. The homogenate was centrifuged at 4000 rpm for 10 min, and the supernatant was taken for the detection of indicators. The activity/level of total protein (TP, A045-2), total cholesterol (TC, A111-1-1), triglycerides (TG, A110-1-1), total superoxide dismutase (T-SOD, A001-1-2), glutathione (GSH, A006-2-1), malondialdehyde (MDA, A003-1-2), acid phosphatase (ACP, A060-2), and alkaline phosphatase (AKP, A059-2) were assayed according to the instructions (Nanjing Jiancheng Bioengineering Institute, Nanjing, China). In addition, Interleukin-1beta (IL-1β, ml07132) and tumor necrosis factor alpha (TNF-ɑ, ml07310) were determined using enzyme-linked immunosorbent assays (ELISA, Shanghai Enzyme-linked Biotechnology Co., Ltd., Shanghai, China).

### 4.5. Untargeted Metabolomic Analysis

Metabolome analysis of *E. sinensis* muscle was performed according to the method described by Xue et al. [51]. Nine samples were selected for each group, and three samples were mixed and accurately weighed. Metabolites were extracted using a pre-cooled extraction solution and freeze-dried (Wonbio-96c, Shanghai Wanbo Biotechnology Co., Ltd., Shanghai, China). Following ultrasonic extraction, the samples were centrifuged to obtain the supernatant. Equal volumes of supernatant were mixed to form a quality control (QC) sample for assessing the reproducibility of the analysis. Finally, the samples were analyzed by LC-MS (UHPLC-Q Extractive system from Thermo Fisher Scientific, Waltham, MA, USA). The raw data were processed by filtering, removing missing values, and filling in blank values. Mass spectral peaks were normalized using the sum normalization method, and variables with relative standard deviation (RSD) > 30% in the QC samples were excluded and log10 processed to finally obtain the standard data for subsequent analysis. The data were subjected to principal component analysis (PCA) using R language (Version 1.6.2). Differential expressed metabolites (DEMs) were identified by variable importance in projection (VIP) and Student’s *t*-test. DEMs were annotated by KEGG database to identify key metabolic pathways.

### 4.6. Untargeted Lipidomic Analysis

Lipidome analysis was performed with reference to the method described by Chen et al. [15]. Nine muscle samples were taken from each group and three samples were mixed. Samples were accurately weighed, and processed for metabolite extraction. After low-temperature ultrasonic extraction and centrifugation, the supernatant was collected. QC samples were prepared for data stability analysis, and samples were analyzed by LC-MS (UHPLC-Q Extractive system from Thermo Fisher Scientific). Data were processed with Progenesis QI software (v2.0) (Waters Corp., Milford, MA, USA) and metabolites were identified using the Human Metabolome Database (HMDB) and Metlin databases. The data were normalized and log10 processed to generate final datasets. Differential lipid metabolites (DLMs) were analyzed using the KEGG database to uncover key signaling pathways and functions.

### 4.7. Transcription Level Analysis

For RNA extraction, nine muscle tissues from each group were homogenized with RNAiso Easy and then centrifuged with Solution H (Takara, Dalian, China). RNA was precipitated by centrifugation, washed with 75% ethanol and resuspended in RNase-free water. The RNA concentration and absorbance (A260/280) were measured, then RNA was treated to remove genomic DNA and reverse transcribed into cDNA using the PrimeScript™ FAST RT Kit (Takara). Finally, the transcript levels of the target genes were determined using the TB Green™ Premix Ex Taq™ II kit (Takara). β-actin was used as an internal reference gene to normalize the gene expression level. The primers were synthesized by Shanghai Generay Biotechnology Co., Ltd. (Shanghai, China), and related information is provided in Table S2.

### 4.8. Statistical Analysis of Data

The levels of gene expression were quantified using the 2^−ΔΔct^ method. Statistical differences between groups were analyzed by Student’s *t*-test and expressed as mean ± standard error of the mean (SEM). The Pearson correlation analysis between the metabolites and the genes was carried out using the SPSS software (version 26.0). * *p* < 0.05, ** *p* < 0.01.

## 5. Conclusions

The present study reveals the beneficial effects of formulated dietary substitution of trash fish and its molecular mechanisms in fattening female crabs. The formulated feed can inhibit muscle cell inflammation, apoptosis and autophagy, thus enhancing immune defense, and is also involved in regulating the balance of lipid and amino acid metabolism and promoting protein synthesis. Metabolomic and lipidomic analyses revealed significant enrichment in pyrimidine and sphingolipid metabolism, particularly sphingolipid metabolism, which is involved in maintaining muscle metabolic homeostasis through the integration of immune and inflammation-related pathways. These findings provided new metabolic intervention targets for crustacean nutritional studies and a theoretical basis for the development of efficient and environmentally friendly aquafeed formulations.

## Data Availability

Data will be made available on request.

## Supplementary Materials

The following supporting information can be downloaded at: https://www.mdpi.com/article/10.3390/ijms26157562/s1.
