# Supplementary material for: Sphingolipid Metabolism Remodels Immunity and Metabolic Network in the Muscle of Female Chinese Mitten Crab (Eriocheir sinensis)
Source: Int J Mol Sci. 2025 Aug 5;26(15):7562. doi: 10.3390/ijms26157562 (PMC12347812; doi:10.3390/ijms26157562)
Supplement: Supplementary file 1 [file ijms-26-07562-s001.zip › ijms-3695408-supplementary.pdf]

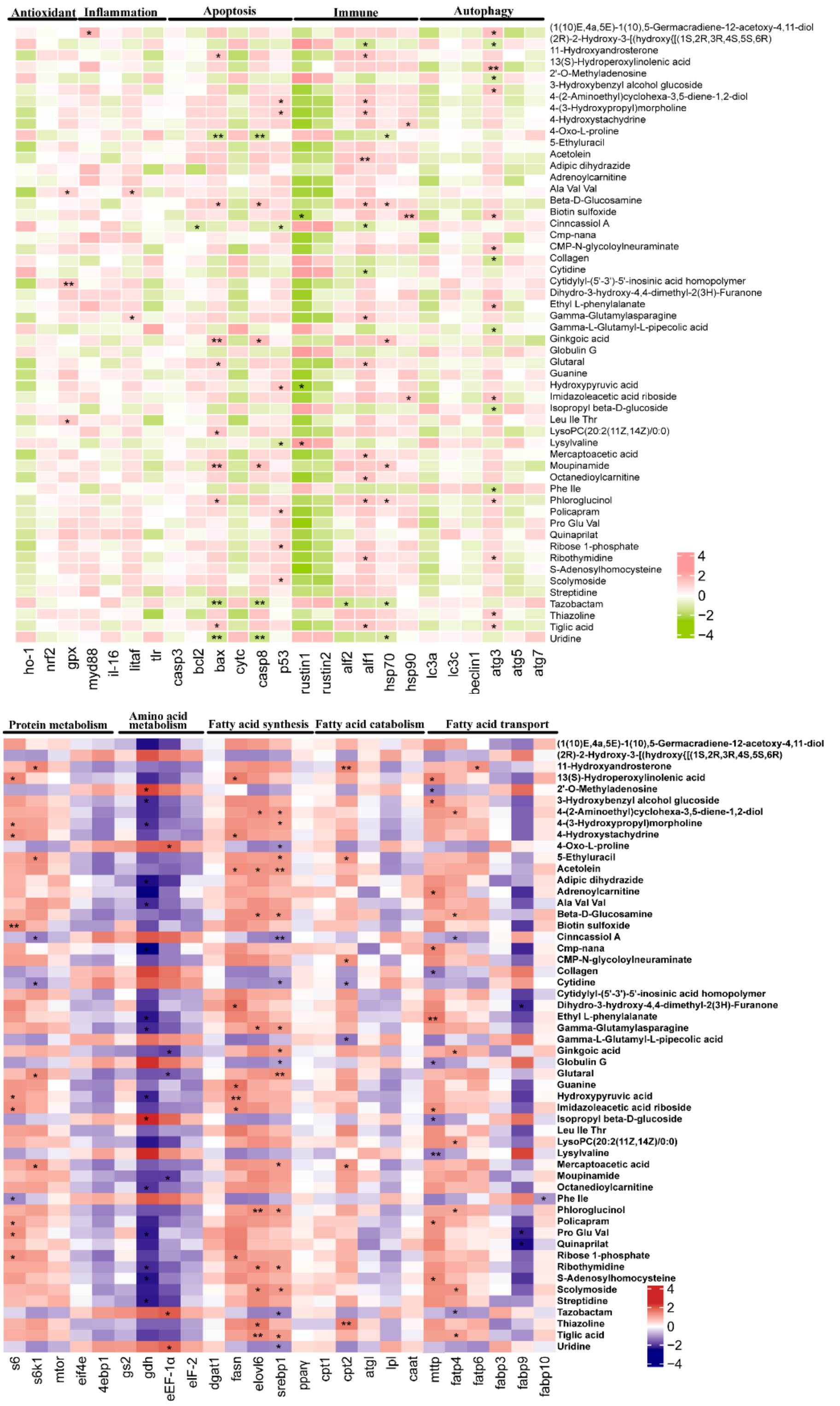

Figure S1. Functional analysis of differential metabolites.

Correlation analysis of DEMs with physiological health and metabolism-related genes. * $P \leqslant 0.05$ and ** $P \leqslant 0.01$.

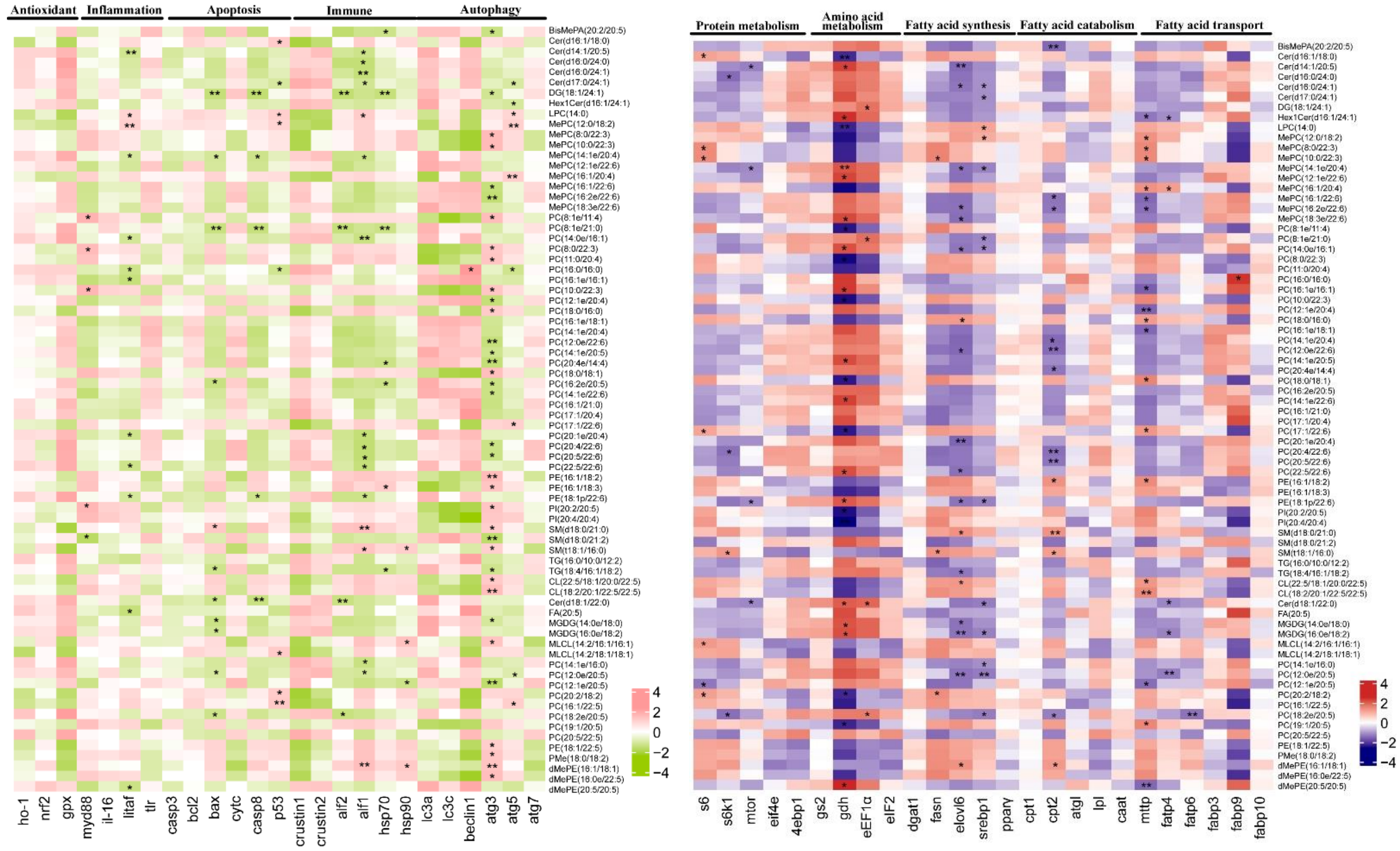

Figure S2. Functional analysis of differential lipid metabolites.

Correlation analysis of DLMs with physiological health and metabolism-related genes.

* $P < 0.05$ and ** $P < 0.01$.

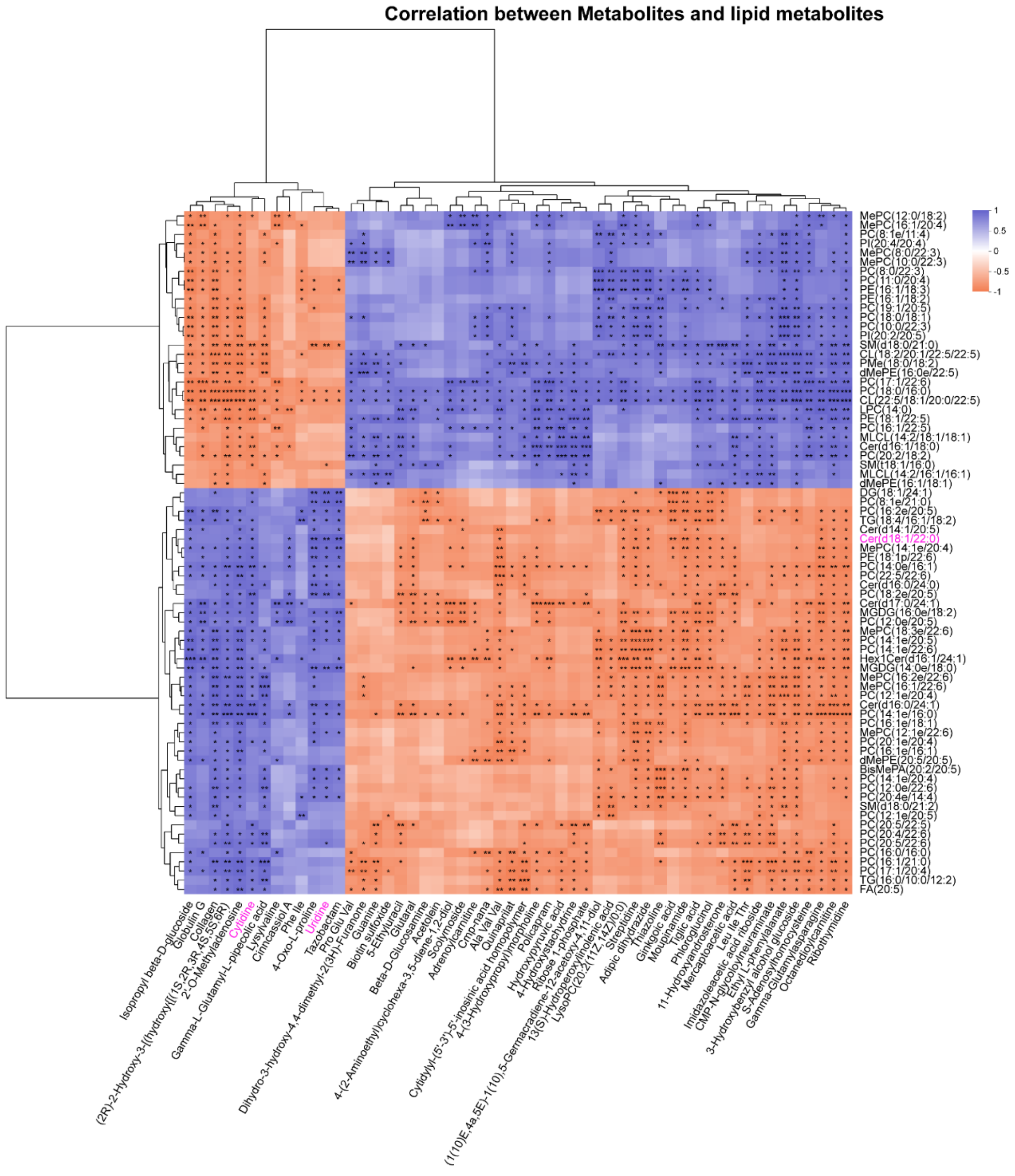

Figure S3 Correlation analysis of metabolites

The right side shows the differential lipid metabolites, and the bottom side shows the differential metabolites. Different colors represent the magnitudes of the correlation coefficients between the properties. The asterisk represents the magnitude of the significance p value, * 0.01 < $P \leqslant 0.05$, ** 0.001 < $P \leqslant 0.01$, *** $P \leqslant 0.0001$.

Table S1 Ingredients and proximate composition of basic feed (dry matter basis)

Ingredients/%	FEED	Nutrition value (% / dry matter)	FEED
Fishmeal	36.00	Dry matter, DM	91.76
Chicken meal	10.00	Crude protein, CP	44.78
Soybean meal	9.10	Ether extract, EE	11.88
Peanut meal	5.00	Ash	7.68
Chicken blood globulin powder	4.00		
Pig blood globulin powder	3.00		
Sesame seed meal	5.00		
Pregelatinized starch	17.00		
Soybean oil	4.50		
Soybean phospholipid oil	2.00		
Sodium chloride (NaCl)	0.20		
Choline Chloride (50%)	0.50		
Monocalcium phosphate	1.00		
Vitamin and minal premix	0.70		
Zeolite powder	2.00		
Total	100.00		

All ingredients are provided by fish disease and nutrition department of Freshwater Fisheries Research Center of Chinese Academy of Fishery Sciences.

Table S2 Primer sequences for target genes for RT-PCR analysis

Gene	Primer sequence (5' — 3')	Gene	Primer sequence (5' — 3')
β-actin	F: TGCGAGACATCAAGGAAAAG<br>R: GGAAGGAAGGCTGGAAGAGT	Atg3	F: CACTTGGTTCATCACTGCCC<br>R: GCGATACTCCATTTGTTTGC
Ho-1	F: TCTAGCCGTAGTCGAAGTTGAAG<br>R: TAACTTTGAAGGTCACCACAGCA	Dgat1	F: AACCGGAAGATCCCTCAGGA<br>R: GGGATCCTGATGGGTGCAAA
Nrf2	F: ACCACAGAAATGAACCAAACCAC<br>R: GTCAGGACTAAGGGAAGACACTG	Fasn	F: ATCCTGACCTCTGAGGACCC<br>R: ATCCCCACTACCCTCAGCAT
Gpx	F: GACTACACCCCAGTGTGCACCA<br>R: TGATCCAGCCATTGTGATCCTC	Elovl6	F: TGAGAAGCGGCAATGGATGAAG<br>R: TGGAGAAGAGGGCCAGGAAGAC
Crustin1	F: GCTCTATGGCGGAGGATGTCA<br>R: CGGGCTTCAGACCCACTTTAC	Srebp-1	F: TCTTCACACCCTCTGGACGC<br>R: CCAAGGTTGTAATGGCACGC
Crustin2	F: GCCCACCTCCCAAACCTAT<br>R: GCAAGCGTCACAGCAGCACT	Pparγ	F: GGCTCTACGCCATCTGAACA<br>R: TCGGGCCACTTCATCTAGGA
Alf1	F: GCTGGCTGGACCGGATTATT<br>R: ATCACACGGGTGTTGCAGAT	Cpt1	F: ATGGAGGTGATTGAGCGAAG<br>R: GATGACCCCTTTCCCAAACT
Alf2	F: TGTCACCCCGCCTCATTAAG<br>R: GTCAGAGACTCCCCCTGGAT	Cpt2	F: AGGACAAGGCAAACAAGCAC<br>R: CTTGGCTGGATTGAGGTGAT
Hsp70	F: TCCCAGCGTACTTTAACGATTCA<br>R: TCGTAGAACATTTAGTCCCGCAA	Atgl	F: TGTAGCAAGCAGAGGAAGGA<br>R: TGCATGAACCTCAGATACGC
Hsp90	F: TCACCAACGACTGGGAGGAT<br>R: CAGGAAGAGGAGTGCCCTGA	Lpl	F: AACTTCCGTCATTGGGTCAC<br>R: ATCACAGGCACAGGAATGGT
Myd88	F: GCCATCGCAGTCGCCAAGTT<br>R: GGCATCCTGTTCATCCAGTTCTGAC	Mttp	F: TAGGACAAGCAGGACTTTCCTCA<br>R: CCACATCCACAAACACATCAACA
Il-16	F: AGAGGTTGTTCTTGTGCTGTCC<br>R: ACGAGGGTAATGGTGAATGGAG	Caat	F: CATCAAGAGCCAGGAGCCCA<br>R: CTTCAACAGCAGCCCGCAAA
Litaf	F: TAAAGGCAAGGGAGGCTTCG<br>R: GAATGGAGCTTGAGGTGGCA	Fatp4	F: GACGGCAGACACGGAAAGAGA<br>R: CAGGTGGAGGCAAGCAAACTC
Tlr	F: CTCCTTCACCTGCCCTAACTGCT<br>R: CTCCAGTTTGTATTGCTGTGCGAAA	Fatp6	F: TGATGGGAAGGCAGGAATGG<br>R: TGCGGATGAAGCGAGGTACA
Casp3	F: CATTCGCCAGCCTTGCCCACA<br>R: TCTGTCGTGGTTCCTTGTAGCCTT	Fabp3	F: CCACCGAGGTCAAGTTCAAGC<br>R: TCACACCATCACACTCCGACAC
Casp8	F: CATGGTGATGAGAATGAC<br>R: TTGGATGAAGTAGAGACG	Fabp9	F: GCCGCACCTCAACTCCACTACAA<br>R: ATCACCAGTCCCACACCCAAAGC
Bcl2	F: ATAAGGTGGTTCGCTCCGTC<br>R: TTAACACAGTCCGAGGCCAG	Fabp10	F: TGCTGATTGGCTCAGTGCTGTG<br>R: CGTGGTCTTGATGACGATGTCG
Bax	F: GTCAGTGAACCTCAGCTGCAT<br>R: CACAGCCACATCACCCACGAA	S6	F: TTCCGAGGGTGAACAAGACG<br>R: CTGGCCCATACGCTTCTCAT
Cytc	F: GGCCAAGGATGTGTCAGTGT<br>R: AGTACGGACCACTTGTGTCG	S6k1	F: TCAATAGCGTCGTCATCG<br>R: CCCTGCGTGTAGTGGTTG
Lc3a	F: ACGTCACGATGGGAGAACTG<br>R: GTGGTGGTGCTCGTAAACCT	mTOR	F: AGAAGCTGCATGACTGGGAC<br>R: CGGTCACACGACACACTGTA
lc3c	F: CACGTTGCCTATCCTCGACA	Elf4e	F: CAAGGCTGAGCAGGACTTCA

Gene	Primer sequence (5' — 3'）	Gene	Primer sequence (5' — 3'）
	R: GTCATCGTCCCTACACTCGC		R: AGCTGATCCAGGTCACAAGC
Beclin1	F: GCCCATATACTGTGGCGAGG	4ebp1	F: CAAGGCTGAGCAGGACTTCA
	R: CCAGGTCAAAGAGCCCAGTT		R: AGCTGATCCAGGTCACAAGC
p53	F: TCGACATGGAAGGGAAGCAC	Gs2	F: TGGAACTTTGACGGCTCTTC
	R: CTGACTTCAAACGGCACAGC		R: TGGAACTTTGACGGCTCTTC
Atg5	F: ACCAGCAGGACGCAGAGATGT	Gdh	F: CCCGCAACTACTCCATCAAT
	R: GTGTGAGAAGTGTGCCGTGAGG		R: GCGTAAGTGTCAGCAATCCA
Atg7	F: TCCGACTTCATCCGAAAATACC	eEf-1α	F: GGTCGGCTACAACCCAACTA
	R: GCACTCAACCCCAAGCCTG		R: CTGCTTGGTGGTTCGATGTT
		eIf-2	F: TGTCCTTCTCACCCAAATCC
			R: ATGGGTATCTCGTTGGTGGA

Table S3 Lipidomic metabolic pathway in Figure 7A

KEGG.name	
Glycerophospholipid metabolism	1
Folate transport and metabolism	2
Sphingolipid metabolism	3
Sphingolipid signaling pathway	4
Lysosome	5
Apoptosis	6
Retrograde endocannabinoid signaling	7
Regulation of lipolysis in adipocytes	8
Glutamatergic synapse	9
Choline metabolism in cancer	10
Long-term depression	11
Ras signaling pathway	12
GnRH signaling pathway	13
Pancreatic cancer	14
Hippo signaling pathway	15
Phospholipase D signaling pathway	16
Fc gamma R-mediated phagocytosis	17
Yersinia infection	18
Axon guidance	19
Chagas disease	20
Toll-like receptor signaling pathway	21
Bacterial invasion of epithelial cells	22
Viral myocarditis	23
Cell adhesion molecules	24
Necroptosis	25
Adipocytokine signaling pathway	26
Insulin resistance	27
Neurotrophin signaling pathway	28
AGE-RAGE signaling pathway in diabetic complications	29
Leishmaniasis	30
Diabetic cardiomyopathy	31
